# Patient Perception of Artificial Intelligence Use in Interpretation of Screening Mammograms: A Survey Study

**DOI:** 10.1148/rycan.240290

**Published:** 2025-04-18

**Authors:** B. Bersu Ozcan, Basak E. Dogan, Yin Xi, Emily E. Knippa

**Affiliations:** ^1^Department of Radiology, University of Texas Southwestern Medical Center, 5323 Harry Hines Blvd, MC 8896, Dallas, TX 75390-8896; ^2^Department of Population and Data Sciences, University of Texas Southwestern Medical Center, Department of Population and Data Sciences, Dallas, Tex

**Keywords:** Breast, Mammography, Artificial Intelligence

## Abstract

**Purpose:**

To assess patient perceptions of artificial intelligence (AI) use in the interpretation of screening mammograms.

**Materials and Methods:**

In a prospective, institutional review board–approved study, all patients undergoing mammography screening at the authors’ institution between February 2023 and August 2023 were offered a 29-question survey. Age, race and ethnicity, education, income level, and history of breast cancer and biopsy were collected. Univariable and multivariable logistic regression analyses were used to identify the independent factors associated with participants’ acceptance of AI use.

**Results:**

Of the 518 participants, the majority were between the ages of 40 and 69 years (377 of 518, 72.8%), at least college graduates (347 of 518, 67.0%), and non-Hispanic White (262 of 518, 50.6%). Participant-reported knowledge of AI was none or minimal in 76.5% (396 of 518). Stand-alone AI interpretation was accepted by 4.44% (23 of 518), whereas 71.0% (368 of 518) preferred AI to be used as a second reader. After an AI-reported abnormal screening, 88.9% (319 of 359) requested radiologist review versus 51.3% (184 of 359) of radiologist recall review by AI (*P* < .001). In cases of discrepancy, higher rate of participants would undergo diagnostic examination for radiologist recalls compared with AI recalls (94.2% [419 of 445] vs 92.6% [412 of 445]; *P* = .20]. Higher education was associated with higher AI acceptance (odds ratio [OR] 2.05, 95% CI: 1.31, 3.20; *P* = .002). Race was associated with higher concern for bias in Hispanic versus non-Hispanic White participants (OR 3.32, 95% CI: 1.15, 9.61; *P* = .005) and non-Hispanic Black versus non-Hispanic White participants (OR 4.31, 95% CI: 1.50, 12.39; *P* = .005).

**Conclusion:**

AI use as a second reader of screening mammograms was accepted by participants. Participants’ race and education level were significantly associated with AI acceptance.

**Keywords:** Breast, Mammography, Artificial Intelligence

*Supplemental material is available for this article.*

Published under a CC BY 4.0 license.

See also commentary by Burkard-Mandel and Joe in this issue.

## Graphical abstract

**Figure:**
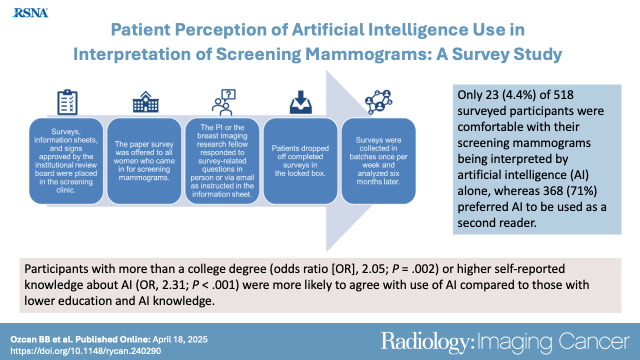

**Summary**

The use of artificial intelligence in screening mammography was supported by patients primarily as a second reader, despite concerns about loss of personal interaction, privacy, transparency, and bias.

**Key Points**

■ Only 23 (4.44%) of 518 surveyed participants were comfortable with their screening mammograms being interpreted by artificial intelligence (AI) alone, whereas 368 (71.0%) preferred AI to be used as a second reader.
■ Participants with more than a college degree (odds ratio [OR] 2.05, 95% CI: 1.31, 3.20; *P* = .002) or higher self-reported knowledge about AI (OR 2.31, 95% CI: 1.51, 3.53; *P* < .001) were more likely to agree with use of AI in interpreting their screening mammogram compared with those with lower education and AI knowledge.

## Introduction

The integration of modern artificial intelligence (AI) in radiology holds the potential to revolutionize medical imaging, improving patient outcomes, daily workflows, and efficiencies (1–5). Among various modalities, screening mammography stands out as highly amenable to AI applications due to its standardized protocols across institutions and high volume of categorical radiologic and pathologic outcome data, which facilitates the development and validation of robust AI algorithms. Unlike conventional computer-aided detection systems that were limited by the need for human-derived features, modern AI systems use deep learning to autonomously identify and learn from complex patterns in large datasets, enhancing diagnostic accuracy and efficiency. However, modern AI use in breast imaging has not been widely adopted due to several challenges that extend far beyond the development and evaluation phases such as concerns surrounding data privacy, security, algorithm transparency, bias, and ethical issues (6,7). Although these challenges to clinical implementation have previously been identified and various mitigating strategies have been proposed, the patient’s perspective and acceptance are frequently overlooked.

A previous qualitative study (8) has explored patients’ viewpoints on AI use in radiology, highlighting areas of concern such as proof of technology, procedural knowledge, competence, efficiency, personal interaction, and accountability. Six domains derived from this study were later used to develop a standardized questionnaire by Ongena et al (9). Patients have expressed a desire to be well informed regarding how and which imaging data will be used and the need for regulatory frameworks before trusting AI. Another finding was the patients’ need for human interaction, mainly when communicating their results (9). Overall, this study revealed that patients were not optimistic about using AI in radiology, at least in the predetermined six domains (9).

In two more recent breast imaging–specific studies (10,11), researchers surveyed women to assess their attitudes toward using AI to interpret screening mammograms. The results of both studies indicated that patients support using AI to assist radiologists in interpreting their mammograms but not as the sole reader. Regarding accountability in the case of a diagnostic error, there was no consensus on whether the radiologist or the company (ie, the developer of the AI system) would be responsible. The majority (52.8%, 418 of 800) agreed that both the radiologist and the developer should be held accountable for the error (11). Interestingly, respondents who were neutral about holding the radiologist responsible were less likely to value personal interaction (10).

Successful implementation of AI in breast imaging—and radiology in general—necessitates careful consideration of patients’ perceptions of its utility, because they are one of the most important key stakeholders in the process. Patients’ acceptance of AI implementation in mammography screening programs in the United States and the factors associated with a more favorable approach toward AI implementation in this context remains uncertain. We hypothesized that patient demographics, education level, and prior screening recall may correlate with their acceptance of AI implementation in mammography screening programs. In our study, we aimed to better understand patients’ opinions and concerns regarding the use of AI to interpret screening mammograms and to identify the factors associated with a more positive attitude toward acceptance.

## Materials and Methods

The present study was an institutional review board–approved, Health Insurance Portability and Accountability Act–compliant prospective survey study with a waiver of consent given that no protected health information would be collected as part of this study.

### Study Participants

**Figure 1:**
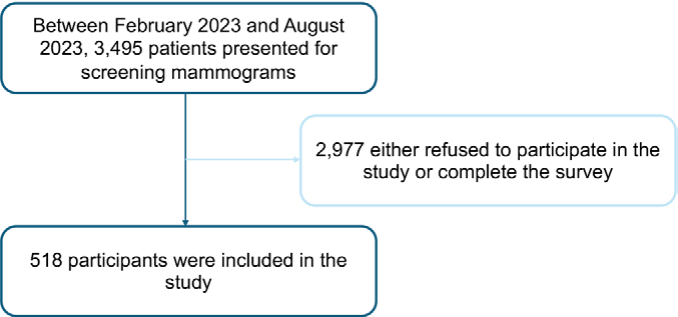
Study flowchart of participant inclusion and exclusion.

According to the American Association for Public Opinion Research Guidelines, a 29-question survey (available in English or Spanish; Figs S1, S2) was offered to patients who presented for screening mammography in our breast cancer screening clinic from February 2023 to August 2023 (12). All patients who presented for screening mammography at our hospital were included in the study sample and offered to complete the survey by a breast imaging research fellow or the front desk staff when available; when no staff were available, posted signage with study information was available in the waiting area. Patients were excluded only if they refused to participate in the study or complete the survey (Fig 1).

### Study Workflow and Survey

**Figure 2:**
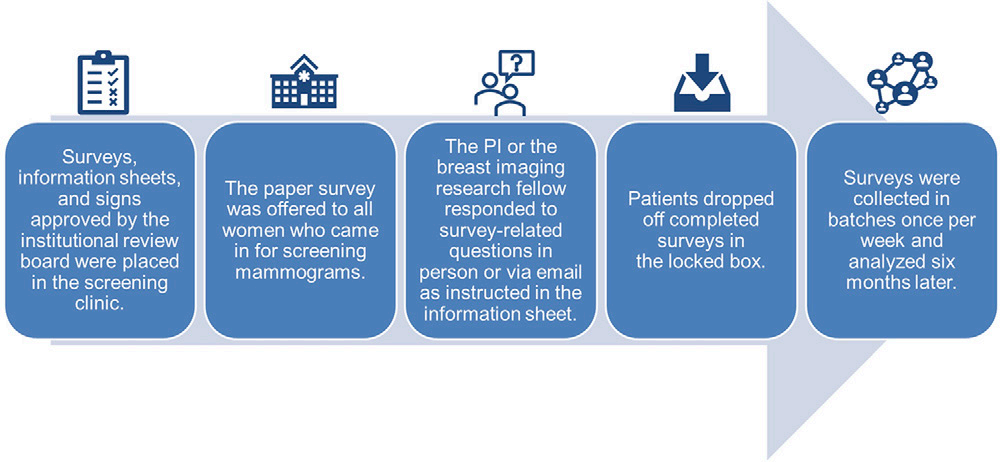
Schematic of the workflow. PI = principal investigator.

The surveys were readily accessible in the clinic and accompanied by informative signage. Both verbal and written information regarding the study’s anonymity and voluntary participation were provided. Each patient received a cover letter (Figs S3, S4) containing details such as the researchers’ names and contact information, the objectives of the study, any possible benefits or risks associated with the study, and the handling of the information provided. Patient questions were answered by the principal investigator of the study or the breast imaging research fellow in person or via email, as instructed in the cover letter. The completed anonymous surveys were deposited in locked collection boxes. Figure 2 provides a schematic of the workflow.

Survey questions assessed six domains—proof of technology, procedural knowledge, competence, efficiency, personal interaction, and accountability—that were derived from a previous qualitative study (8). Closed-ended questions and a five-point Likert-type scale were used to understand patient perceptions regarding AI use. Demographic and clinical information, including age, race and ethnicity (self-reported), native language, education level, annual income, menopausal status, the number of first-degree relatives with breast cancer, age at first mammography screening, past breast cancer diagnoses, and history of abnormal mammograms and/or breast biopsies were also collected.

### Statistical Analysis

Continuous variables were reported as means and SDs, and categorical variables were reported as frequencies and percentages, including missing data in each category. We compared the rate of respondents who requested a radiologist review after an AI-reported versus a radiologist-reported abnormality, as well as assessed the likelihood of diagnostic follow-up for radiologist recalls compared with AI recalls using the McNemar test. For these comparisons, responses were categorized as “Not at all” versus “Probably or Definitely.” We performed univariable logistic regression to evaluate the association of participants’ characteristics with survey responses. Odds ratios (ORs) with 95% CIs were reported to estimate the association between a specific response and participants’ characteristics. In addition, multivariable logistic regression was used to assess whether the association between response and participants’ characteristics persisted after adjusting for age, race, level of education, and participants’ self-reported understanding or knowledge of AI. A *P* value of less than .05 was considered statistically significant. All analyses were performed in R version 4.2.2 (R Core Team); the tidyverse and janitor packages were used to prepare the data, including data wrangling, cleaning, and variable formatting, and the gtsummary package was used to generate tables of ORs and in SAS version 9.5 (SAS Institute).

## Results

### Participant Characteristics

All surveys collected during the study period were included. Of the 3495 patients who underwent screening mammography in our screening clinic between February 2023 and August 2023, 518 (14.82%) agreed to participate in the study (Fig 1). The distribution of age and race and ethnicity was similar between respondents and the overall patient sample, and we found no differences for age (*P* = .66) and race and ethnicity (*P* = .48) (Table S1).

Responses from 518 participants were analyzed. Of these, 7.92% (41 of 518) reported undergoing their initial screening mammography. The majority of participants (377 of 518, 72.8%) were between 40 and 69 years old, identified as non-Hispanic White (262 of 518, 50.6%), and had attained college-level education or higher (347 of 518, 67.0%). One hundred fifty (29.0%) participants were premenopausal, and 141 (27.2%) had at least one first-degree relative with breast cancer. Regarding medical history, 5.21% (27 of 518) of the participants were previously diagnosed with breast cancer, 35.7% (185 of 518) reported abnormal mammography results, and 22.8% (118 of 518) had undergone a breast biopsy. The majority of participants spoke English (418 of 518, 80.7%) as their primary language, and 42.9% (222 of 518) had a yearly income of more than $100 000. Table 1 shows a summary of patient demographics and clinical characteristics.

**Table 1:**
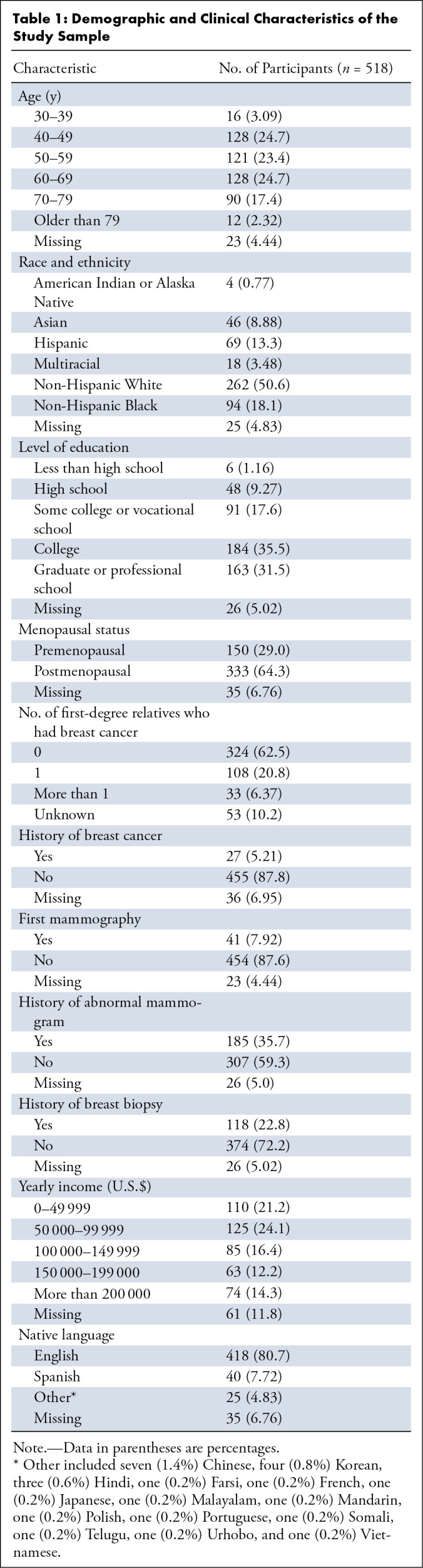
Demographic and Clinical Characteristics of the Study Sample

### Survey Responses

Participants self-reported AI knowledge rates were as follows: 230 (44.4%) indicated that they know “A little bit” about AI, whereas only one (one of 518, 0.19%) participant considered themselves an “AI expert,” and 166 (32.1%) have no knowledge of AI. Consent was deemed necessary by 74.1% (384 of 518) before using AI for mammogram interpretation. Seventy-one percent (368 of 518) of the participants preferred AI to be used as a second reader, and 4.44% (23 of 518) were comfortable with their mammograms being interpreted by AI alone. Turnaround time preferences were distributed, with 19.5% (101 of 518) desiring immediate AI results and 57.1% (296 of 518) willing to wait several hours to a few days for a radiologist’s review. A total of 359 (69.3%) participants responded to both questions regarding additional reading requests following AI versus radiologist recall. After an AI-interpreted abnormal screening, 88.9% (319 of 359) requested radiologist review before scheduling a follow-up examination, compared with 51.3% (184 of 359) of radiologist recall reviews by AI (*P* < .001). Four hundred forty-five (85.9%) participants responded to both questions about their likelihood of agreeing to schedule a diagnostic examination when there is a discrepancy between AI versus radiologist assessment recalls, though the difference was not statistically significant (94.2%, 419 of 445 vs 92.6%, 412 of 445; *P* = .20). When assessing AI’s efficacy compared with radiologists, 5.21% (27 of 518) believed AI to be “much worse,” 21.0% (109 of 518) said “worse,” 43.4% (225 of 518) said “same,” 13.5% (70 of 518) said “better,” and 1.16% (six of 518) said “much better.” Regarding accountability for missed cancer detections by AI, 57.7% (299 of 518) believed everyone should be accountable, and 14.9% (77 of 518) would hold the AI manufacturer responsible. Concerning data privacy, 29.5% (153 of 518) were moderately concerned, and 35.5% (184 of 518) were very or extremely concerned. Concerns were expressed by respondents regarding AI’s functionality (378 of 518, 73.0%), reduced radiologist-patient interaction due to AI (397 of 518, 76.6%), lack of transparency (384 of 518, 74.1%), and possible bias by AI (327 of 518, 63.2%). Participant responses to each question are provided in Tables 2 and S2.

**Table 2:**
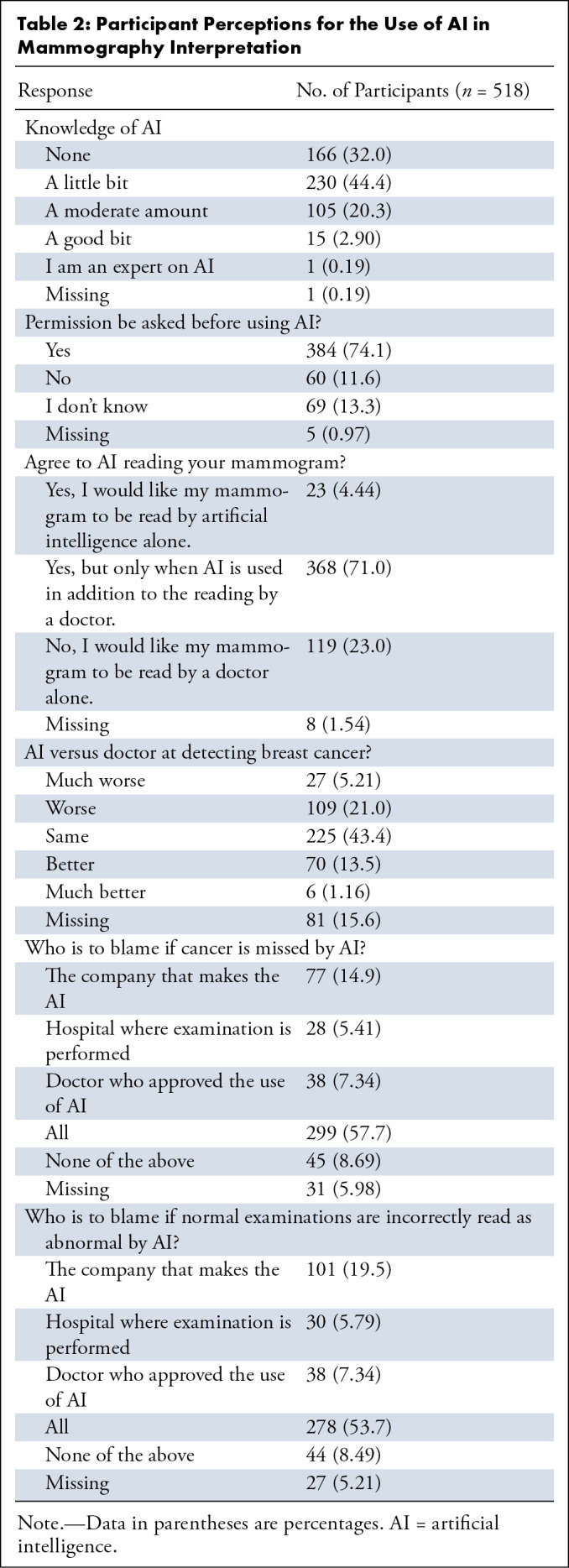
Participant Perceptions for the Use of AI in Mammography Interpretation

### Association of Participant Characteristics and Perceptions of AI Use

Tables 3 and S3 display the association between participant characteristics and perceptions toward AI use in mammogram interpretation.

**Table 3:**
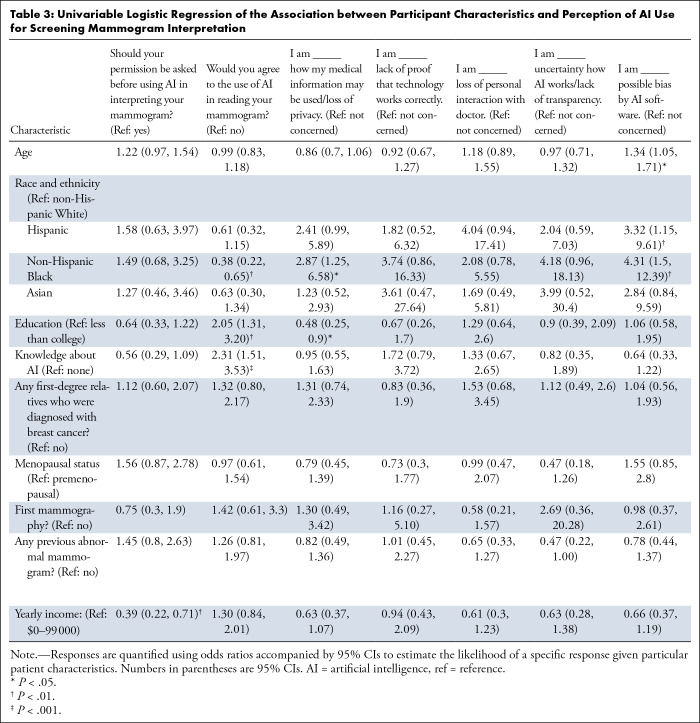
Univariable Logistic Regression of the Association between Participant Characteristics and Perception of AI Use for Screening Mammogram Interpretation

### Should You Be Asked before AI Interprets Mammograms?

Yearly income level was a significant factor in deciding whether permission should be sought before using AI. Participants with a yearly income exceeding $99 000 were less likely to consider permission necessary compared with those with a lower income (OR 0.39, 95% CI: 0.22, 0.71; *P* = .002).

### Would You Agree with AI to Read Your Mammogram?

Compared with non-Hispanic White participants, non-Hispanic Black participants were less likely to agree with the use of AI (OR 0.38, 95% CI: 0.22, 0.65; *P* = .005), whereas we found no evidence of a difference between other race and ethnicity groups (Hispanic: OR 0.61, 95% CI: 0.32, 1.15, *P* = .12; Asian: OR 0.63, 95% CI: 0.30, 1.34, *P* = .20). Participants with more than a college degree (OR 2.05, 95% CI: 1.31, 3.20; *P* = .002) or a higher knowledge about AI (OR 2.31, 95% CI: 1.51, 3.53; *P* < .001) were approximately two times more likely to agree with AI usage in reading their mammogram. Factors such as age (OR 0.99, 95% CI: 0.83, 1.18; *P* = .89) and annual income (OR 1.30, 95% CI: 0.84, 2.01; *P* = .25) and clinical characteristics including having a first-degree relative diagnosed with breast cancer (OR 1.32, 95% CI: 0.80, 2.17; *P* = .28), previous mammography screenings (OR 1.42, 95% CI: 0.61, 3.30; *P* = .42), or not having a history of abnormal mammograms (OR 1.26, 95% CI: 0.81, 1.97; *P* = .31) did not significantly influence patients’ acceptance of AI in mammogram interpretation.

### Re-evaluation Requests: AI versus Radiologist Recalls

Participants with first-degree relatives diagnosed with breast cancer were significantly more likely to seek a radiologist’s re-evaluation after an AI-detected abnormality (OR 3.25, 95% CI: 1.24, 8.5; *P* = .02), whereas the same group did not differ from their counterparts in requesting an AI re-evaluation after a radiologist-detected abnormality (OR 1.59, 95% CI: 0.99, 2.55; *P* = .05). Compared with the non-Hispanic White participants, participants identifying as Asian (OR 4.14, 95% CI: 1.85, 9.22; *P* < .001) were significantly more likely to opt for an AI re-evaluation. We found no evidence of a difference among non-Hispanic Black (OR 0.75, 95% CI: 0.42, 1.33; *P* = .30) or Hispanic (OR 1.57, 95% CI: 0.87, 2.85; *P* = .13) groups. Participants who had undergone mammography before were significantly more likely to request an AI review after a radiologist detected abnormality (OR 3.11, 95% CI: 1.35, 7.16; *P* = .008).

### Discrepancies between AI and Radiologist Callbacks: Agreement on Diagnostic Mammogram Scheduling

Compared with non-Hispanic White participants, participants identifying as non-Hispanic Black (OR 0.30, 95% CI: 0.12, 0.79; *P* = .01) or Asian (OR 0.22, 95% CI: 0.07, 0.65; *P* = .01) were significantly less inclined to agree to a follow-up diagnostic examination after AI reported a normal result. We found no evidence of a difference between non-Hispanic White and Hispanic participants.

Participants with a history of abnormal mammograms were more likely to agree to scheduling a diagnostic examination upon receiving an abnormal report from either a radiologist-reported abnormality (OR 4.98, 95% CI: 1.47, 16.85; *P* = .01) or an AI-reported abnormality (OR 10.08, 95% CI: 2.38, 42.76; *P* = .002).

### Do Patients Think AI Is Better, the Same, or Worse than a Radiologist at Detecting Breast Cancer?

We found no evidence of a difference in the perception of AI’s diagnostic accuracy as compared with a radiologist’s, based on age (OR 0.92, 95% CI: 0.77, 1.09; *P* = .32), race and ethnicity (Hispanic: OR 1.00, 95% CI: 0.54, 1.86, *P* = .99; non-Hispanic Black: OR 0.85, 95% CI: 0.49, 1.49, *P* = .58; Asian: OR 0.62, 95% CI: 0.31, 1.23, *P* = .17; all compared with non-Hispanic White), educational attainment (OR 0.85, 95% CI: 0.54, 1.35; *P* = .50), or menopausal status (OR 1.22, 95% CI: 0.78, 1.89; *P* = .39). Similarly, we found no evidence of a difference between higher knowledge about AI (OR 1.44, 95% CI: 0.93, 2.21; *P* = .10), participants with a family history of breast cancer (OR 1.26, 95% CI: 0.78, 2.01; *P* = .34), higher income (>$99 000: OR 0.95, 95% CI: 0.62, 1.45; *P* = .80), or those without a previous abnormal mammogram (OR 0.93, 95% CI: 0.61, 1.43; *P* = .74) in their trust in AI over radiologist for detecting breast cancer.

### Accountability for AI Errors: Attribution in Cases of False Negatives and False Positives (Reference: The Company That Makes AI or All Entities Should Be Blamed)

In the context of AI-associated false negatives, participants with previous abnormal mammograms were significantly more likely to hold the hospital or the endorsing radiologist accountable than the AI developers or all entities combined (OR 1.71, 95% CI: 1.11, 2.64; *P* = .02). For false positives caused by AI, Hispanic participants were notably more likely to assign responsibility to the AI developers or collectively to all involved parties (OR 0.41, 95% CI: 0.19, 0.91; *P* = .03).

### Concerns about Medical Information Use, Privacy, AI Accuracy, Radiologist Interaction, AI Transparency, and Possible Bias

Compared with non-Hispanic White, non-Hispanic Black participants were significantly more concerned regarding privacy and the potential misuse of medical information (OR 2.87, 95% CI: 1.25, 6.58; *P* = .03) and possible bias (OR 4.31, 95% CI: 1.50, 12.39; *P* = .005). Similarly, Hispanic participants expressed a higher concern regarding possible bias (OR 3.32, 95% CI: 1.15, 9.61; *P* = .005). Less concern about handling medical data were observed in participants with higher educational attainment versus those with a high school education or less (OR 0.48, 95% CI: 0.25, 0.90; *P* = .02). Age was a significant factor, with an increase in age associated with increased concern of AI bias (OR 1.34, 95% CI: 1.05, 1.71; *P* = .02).

### Multivariable Analysis

Multivariable logistic regression analysis was conducted to assess the association between participants’ characteristics and acceptance of AI use after adjusting for age, race, level of education, and knowledge about AI (Tables 4, S4). Participants with a first-degree relative diagnosed with breast cancer had a significantly higher likelihood of seeking re-evaluation from a radiologist or AI after an abnormal finding by AI (OR 3.02, 95% CI: 1.13, 8.06; *P* = .03) or by a radiologist (OR 1.91, 95% CI: 1.14, 3.21; *P* = .01). Additionally, a history of abnormal mammograms significantly influenced preferences for follow-up examinations upon diagnostic discrepancies, with prior mammography experience increasing the likelihood of seeking a follow-up for abnormalities reported by either radiologist (radiologist reports abnormal, AI reports normal: OR 4.41, 95% CI: 1.26, 15.49; *P* = .02) or AI (radiologist reports normal, AI reports abnormal: OR 10.69, 95% CI: 2.47, 46.29; *P* = .002).

**Table 4:**
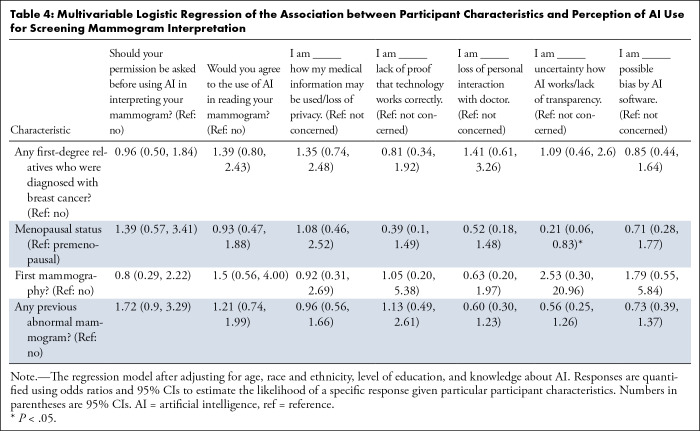
Multivariable Logistic Regression of the Association between Participant Characteristics and Perception of AI Use for Screening Mammogram Interpretation

## Discussion

We aimed to explore patient perceptions regarding the use of AI in interpreting screening mammograms. With 518 female participants, the study offers insights into varied viewpoints influenced by demographics, personal medical history, and awareness of AI. Our study showed that the use of AI in screening mammography was supported by participants, mainly as a second reader (368 of 518, 71.0%), despite concerns about loss of personal interaction, privacy, lack of transparency, and bias.

Most participants expressed a preference for AI use alongside a radiologist’s review. This aligns with findings from previous studies, which also highlighted patients’ inclination toward a collaborative approach between AI and health care professionals in mammography interpretation (10,11). In the survey study by Ongena et al (10), which involved 922 patients, a significant majority (77.8%) expressed reservations about the fully independent use of AI for interpreting screening mammography. Another study (11), involving 800 patients, found that 94% of patients believed radiologists should always generate their own mammogram reports, whereas 77% supported the idea of AI serving as a second reader. This preference underscores the importance of maintaining human oversight in AI applications and a broader desire for trust, empathy, and accountability in health care—qualities patients often associate with human providers rather than machines. The role of the radiologist is not only technical but also relational, offering patients reassurance and the opportunity for dialogue. Integrating AI as an adjunct rather than a replacement can meet patient desires for continued humanism in medicine and address concerns about depersonalization in health care.

Our results indicate that participants’ willingness to rely on AI differs based on various demographic factors. Specifically, individuals with higher educational attainment (OR 2.05, 95% CI: 1.31, 3.20; *P* = .002) or more extensive self-reported knowledge (OR 2.31, 95% CI: 1.51, 3.53; *P* < .001) of AI were more open to the use of AI without additional permission requests. Moreover, participants with education attainment above college demonstrated less concern about privacy loss (OR 0.48, 95% CI: 0.25, 0.90; *P* = .02) compared with their counterparts with lower education attainment. This might reflect a broader understanding of technology among these groups, suggesting that educational interventions could enhance AI acceptance. The diminished apprehension about privacy among these individuals likely arises from a nuanced comprehension of data protection mechanisms within AI frameworks. These insights underscore the importance of transparent and effective communication regarding AI’s privacy measures, ensuring all patients, regardless of educational background, are well informed to give consent and sustain confidence in these technologies.

Concerns about data privacy and potential AI biases were notably higher among Hispanic (data privacy: OR 2.41, 95% CI: 0.99, 5.89, *P* = .05; bias: OR 3.32, 95% CI: 1.15, 9.61, *P* = .005) and non-Hispanic Black participants (data privacy: OR 2.87, 95% CI: 1.25, 6.58, *P* = .03; bias: OR 4.31, 95% CI: 1.50, 12.39, *P* = .005). These concerns may have contributed to the lower acceptance of AI use among non-Hispanic Black participants (OR 0.38, 95% CI: 0.22, 0.65; *P* = .005). Our findings highlight the critical need to address racial and ethnic disparities within AI health care applications, revealing that participants, like researchers, are concerned about potential biases (13–16). These biases can arise at any stage of the development and deployment life cycle, including problem definition, data selection and curation, model training, deployment, and postdeployment monitoring (17–19). Given that bias at one stage can propagate to subsequent stages, it is crucial for researchers to continually generate and use diverse and representative datasets (17). Moreover, the development and implementation of stringent testing and validation protocols with continuous monitoring and evaluation of model performance, are essential for reducing bias in AI applications (19). To address patient concerns, transparent communication is necessary. By clearly explaining the nature of bias, the measures implemented for testing and detecting bias before deployment, and the steps taken for its mitigation—as well as the safeguards in place for the protection of patient data—we can enhance trust and understanding, thereby fostering a more inclusive and equitable AI health care environment.

After adjusting for age, race and ethnicity, level of education, and knowledge about AI, our analysis suggested that personal experience with breast cancer significantly influences participant responses to AI and radiologist interpretations in mammography. Participants with first-degree relatives diagnosed with breast cancer showed a higher likelihood of requesting additional reviews, either by a radiologist or AI, in cases of reported abnormalities (AI reported abnormality: OR 3.02, 95% CI: 1.13, 8.06, *P* = .03; radiologist reported abnormality: OR 1.91, 95% CI: 1.14, 3.21, *P* = .01). Interestingly, these participants seemed less inclined to schedule diagnostic examinations when either AI or radiologist reported normal results, indicating a degree of trust in either assessment (AI normal: OR 0.98, 95% CI: 0.37, 2.58, *P* = .97; radiologist normal: OR 0.75, 95% CI: 0.33, 1.72, *P* = .50), although this did not reach statistical significance. Conversely, participants with a history of abnormal mammograms were more inclined to agree to schedule diagnostic examination amid discrepancies between AI and radiologist assessments (radiologist recall: OR 4.41, 95% CI: 1.26, 15.49, *P* = .02; AI recall: OR 10.69, 95% CI: 2.47, 46.29, *P* = .002). This highlights the critical role of individual patient histories and experiences in tailoring and applying AI technologies within health care settings.

Our study had some limitations. First, AI is a complex topic, and despite providing a brief explanation in the information sheet, survey materials, and during the survey presentation, it is possible that patients may not have fully understood the concept. This may have influenced their responses. However, we believe that the responses we obtained reflect patients’ opinions in the absence of detailed prior knowledge, thus capturing perspectives representative of the general public. Second, it was conducted in a university hospital in the United States, where most patients (approximately 70%) were covered by private insurance. Thus, our results might vary across different institutions, states, and countries due to differences in insurance coverage and health care accessibility. Additionally, our study focuses exclusively on the screening setting and does not encompass clinical settings, such as for symptomatic patients. Although 6850 screening mammographic examinations were performed at our institution during the study period, the survey was only offered to patients who underwent screening at the dedicated screening-only floor, comprising 3495 (51.02%) of these cases. Patients screened at the diagnostic floor were not offered the survey because this location primarily accommodated same-day readings for those with concurrent appointments or those traveling long distances who preferred immediate results. Screenings on the diagnostic floor were interspersed with diagnostic cases and, due to operational workflow constraints, were excluded from survey distribution. Additionally, we used a hybrid model in which either a research fellow or front desk staff presented the study with a standardized script, depending on their availability. Patients also had access to the survey, which was readily available in the clinic. The availability of staff likely served as a rate-limiting factor for patient recruitment, thereby affecting the response rate. Consequently, the survey response rate among participants who were exposed to the survey opportunity was 14.82% (518 of 3495). Last, we acknowledge that the relatively small sample size and limited demographic diversity in our survey may not fully generalize to the broader population undergoing mammographic screening. Further research is essential to determine whether our findings are generalizable to different health care contexts and patient populations.

In conclusion, our results indicate a cautious optimism among participants about the integration of AI in mammogram interpretation. Although there is an acceptance of AI, especially when used alongside a radiologist, there remain important concerns about its efficacy, potential bias, and the implications for patient– doctor interactions. As AI technology advances, it is necessary to continue engaging with patients to understand their evolving views and address any concerns through education, transparency, and robust AI validation processes.
